# Azilsartan inhibits inflammation-triggered bone resorption and osteoclastogenesis *in vivo* via suppression of TNF-α expression in macrophages

**DOI:** 10.3389/fendo.2023.1207502

**Published:** 2023-09-15

**Authors:** Ziqiu Fan, Hideki Kitaura, Jiayi Ren, Fumitoshi Ohori, Takahiro Noguchi, Aseel Marahleh, Jinghan Ma, Kayoko Kanou, Mariko Miura, Kohei Narita, Angyi Lin, Itaru Mizoguchi

**Affiliations:** ^1^ Division of Orthodontics and Dentofacial Orthopedics, Tohoku University Graduate School of Dentistry, Sendai, Miyagi, Japan; ^2^ Frontier Research Institute for Interdisciplinary Sciences, Tohoku University, Sendai, Miyagi, Japan

**Keywords:** osteoclast, azilsartan, bone resorption, TNF-α, LPS, Angiotensin II type 1 receptor blocker

## Abstract

**Introduction:**

Hypertension is a major risk factor for cardiovascular disease (CVD) and is associated with increased bone loss due to excessive activity of the local renin-angiotensin system (RAS). Angiotensinogen/Angiotensin (ANG) II/Angiotensin II type 1 receptor (AT1R) axis is considered as the core axis regulating RAS activity. Azilsartan is an FDA-approved selective AT1R antagonist that is used to treat hypertension. This study aimed to determine whether azilsartan affects formation of osteoclast, resorption of bone, and the expression of cytokines linked with osteoclastogenesis during lipopolysaccharide (LPS)-triggered inflammation *in vivo*.

**Methods:**

*In vivo*, following a 5-day supracalvarial injection of LPS or tumor necrosis factor-alpha (TNF-α) with or without azilsartan, the proportion of bone resorption and the number of tartrate-resistant acid phosphatase (TRAP)-positive multinucleated cells, which are identified as osteoclasts on mice calvariae were counted. The mRNA expression levels of TRAP, cathepsin K, receptor activator of NF-κB ligand (RANKL), and TNF-α were also evaluated. *In vitro*, the effect of azilsartan (0, 0.01, 0.1, 1, and 10 μM) on RANKL and TNF-α-triggered osteoclastogenesis were investigated. Also, whether azilsartan restrains LPS-triggered TNF-α mRNA and protein expression in macrophages and RANKL expression in osteoblasts were assessed. Furthermore, western blotting for analysis of mitogen-activated protein kinases (MAPKs) signaling was conducted.

**Results:**

Azilsartan-treated calvariae exhibited significantly lower bone resorption and osteoclastogenesis than those treated with LPS alone. *In vivo*, LPS with azilsartan administration resulted in lower levels of receptor activator of RANKL and TNF-α mRNA expression than LPS administration alone. Nevertheless, azilsartan did not show inhibitory effect on RANKL- and TNF-α-triggered osteoclastogenesis *in vitro*. Compared to macrophages treated with LPS, TNF-α mRNA and protein levels were lower in macrophages treated by LPS with azilsartan. In contrast, RANKL mRNA and protein expression levels in osteoblasts were the same in cells co-treated with azilsartan and LPS and those exposed to LPS only. Furthermore, azilsartan suppressed LPS-triggered MAPKs signaling pathway in macrophages. After 5-day supracalvarial injection, there is no difference between TNF-α injection group and TNF-α with azilsartan injection group.

**Conclusion:**

These findings imply that azilsartan prevents LPS-triggered TNF-α production in macrophages, which in turn prevents LPS-Triggered osteoclast formation and bone resorption *in vivo*.

## 1 Introduction

Hypertension is one of the most important risk factors for cardiovascular disease (CVD) and it contributes to the global disease burden. A large number of individuals are impacted by hypertension and according to the Global Burden of Disease research, approximately 3.5 billion persons worldwide had systolic blood pressure in 2015 that was at least 110 to 115 mmHg, which is linked to an elevated risk of ischemic heart disease (IHD), stroke, and renal disease. Moreover, it is anticipated that by 2025, 1.56 billion adults will have hypertension, representing a 60% increase in the global burden of the disease (1–4). In addition to its effects on the cardiovascular system, hypertension negatively affects bone health. Individuals with hypertension may have a higher risk of osteoporosis and fractures, potentially because hypertension can lead to impacts on calcium homeostasis, vascular function, inflammation, and oxidative stress which may change bone structure and function in the body (5–7). Elevated blood pressure causes osteoclast formation and activity and promotes bone resorption (8).

Osteoclastogenesis is initiated by macrophage colony-stimulating factor (M-CSF) and receptor activator of NF-κB ligand (RANKL), which induce hematopoietic monocytes differentiation into osteoclasts (9). M-CSF and RANKL are produced by osteoblasts and regulate the differentiation and activation of osteoclasts. The interaction between RANKL and its receptor, RANK, which is located on the surface of osteoclast precursors, triggers a series of signaling events that lead to the activation and differentiation of osteoclasts. In addition to M-CSF and RANKL, tumor necrosis factor-alpha (TNF-α) was implicated in osteoclastogenesis. TNF-α can promote RANKL production in stromal cells, leading to increased osteoclast differentiation and activation. Additionally, TNF-α can directly induce osteoclast formation by interacting with its receptors on osteoclast precursor cells (10–12). Lipopolysaccharide (LPS) is a major surface antigen found in gram-negative bacteria and is known to strongly promote inflammation and loss of bone tissue through osteolysis (13). Several studies have indicated that LPS-injected mice exhibit osteoclastogenesis and bone resorption (14–16). Moreover, LPS triggers RANKL expression in stromal cells, which promotes osteoclastogenesis. LPS can also activate macrophages to release TNF-α, which further increases the expression of RANKL by osteoblasts (17).

Many studies have shown that hypertension increases the possibility of losing bone mineral density (18–21). Inflammation is closely associated with hypertension, as evidenced by recent animal studies demonstrating elevated plasma levels of proinflammatory cytokines, including C-reactive protein (CRP), interleukin (IL)-6, IL-1β, and TNF-α, in hypertensive animals (22, 23). Numerous clinical investigations have revealed that people with high blood pressure frequently have higher plasma concentrations of proinflammatory cytokines compared to normotensive patients, which is consistent with the findings of animal research (24, 25). Blood pressure and electrolyte balance are tightly controlled by the renin-angiotensin system (RAS). Hypertension is significantly influenced by RAS activation (26, 27). RAS is a hormonal cascade in which renin from the kidney cleaves angiotensinogen from the liver into inactive angiotensin (ANG) I. Circulating ANG I attaches to angiotensin-converting enzyme (ACE), which converts ANG I into ANG II. ANG II binds to type 1 or type 2 receptor, regulating ANG II downstream bioactivities. Angiotensinogen/ANG II/Angiotensin II type 1 receptor (AT1R) axis is considered as the core of the RAS. Local activation of the ANG II/AT1R pathway leads to pathological changes such as vasoconstriction, fibrosis, inflammation, and bone loss. Many tissues, like bone contain a local RAS in addition to the systemic RAS, whose excessive activity increases bone resorption and decreases bone formation (28). ANG II contributes to osteoporosis by suppressing osteoblastic stromal calcification as well as increasing osteoclast bone resorption activity. For the majority of its biological reactions, ANG II uses AT1R as a mediator. Pro-inflammatory chemicals including IL-6 and TNF-α, which encourage bone resorption, is further stimulated by ANG II. Several studies showed angiotensin II receptor blockers (ARB) bind to AT1R and block ANG II, preventing pathological changes (27–31).

Our previous research has shown that salt-sensitive hypertension (SSHTN) negatively affects bone health. Increased production of the pro-inflammatory cytokine TNF-α and excessive bone RAS activation may have contributed to the deterioration of the bone microstructure observed in these hypertensive animals (32).

Azilsartan is an FDA-approved selective AT1 receptor antagonist used to treat hypertension (33). Without increasing the side effects, azilsartan is more effective at its maximum permitted dose than olmesartan or valsartan, both of which are selective AT1 receptor antagonists (34). Azilsartan, an angiotensin receptor blocker, exhibits anti-inflammatory properties. Decreased levels of plasma inflammatory cytokines, just like IL-6 and TNF-α were spotted in the azilsartan treatment group compared to the diabetic group (31). In addition, LPS-triggered gene expression elevation and secretion of MCP-1, IL-6, and IL-1β were dramatically reduced by azilsartan in a dose-dependent manner (35). In addition to its antihypertensive and anti-inflammatory properties, azilsartan may also regulate bone remodeling. In an ovariectomy-induced osteoporosis mouse model, azilsartan treatment could significantly reduce the number of tartrate-resistant acid phosphatase (TRAP)-positive cells in the long bone compared to the vehicle groups (36). In a ligature-induced periodontal rat model, azilsartan treatment could improve bone loss (37). However, despite the known effects of azilsartan on hypertension and inflammation, there is a lack of studies investigating the specific mechanism by which azilsartan reduces inflammation-triggered osteoclastogenesis and bone resorption *in vivo*. Therefore, the top priority of the study was to address this knowledge gap by examining the impact of azilsartan on LPS-triggered osteoclast formation and bone destruction, both *in vivo* and *in vitro*.

## 2 Materials and methods

### 2.1 Reagents and animals

Male C57BL6/J mice that were eight to ten weeks old were acquired from CLEA Japan, Inc. (Tokyo, Japan) and maintained at an animal facility. The Science Animal Care and Use Committee of Tohoku University’s guidelines were followed in full for all animal care and experimental procedures. Each experimental group consisted of four randomly assigned mice. Azilsartan was purchased from Sigma-Aldrich (St. Louis, MO) and dissolved in DMSO before being kept at 4°C. LPS from Escherichia coli were bought from Sigma-Aldrich (St. Louis, MO). As previously mentioned, recombinant murine TNF-α (38) and M-CSF (CMG14-12 cell line) (39) were produced.

### 2.2 Experimental model *in vivo*

*In vivo* study previously showed that subcutaneous injections of 100 μg LPS or 3 μg TNF-α per day into mouse calvariae for five consecutive days successfully triggered osteoclastogenesis (40, 41). The same guidelines, dosages, and administration times were used in this study. The mice were splited into four groups for the experiments and each group received daily administration of phosphate-buffered saline (PBS), LPS (100 µg/day) or TNF-α (3 μg/day) in the presence or absence of azilsartan (100 µg/day), and azilsartan only (100 µg/day).

### 2.3 Histological analysis

All mice were euthanized on the sixth day, and their calvariae were promptly excised and fixed in 4% PBS-buffered formaldehyde. After 3 days of fixation at 4°C, all calvariae were demineralized for 3 days at room temperature with 14% ethylenediaminetetraacetic acid (EDTA). All calvariae were dehydrated in a tissue processor (TP1020; Leica, Wetzlar, Germany) before being embedded in paraffin and sliced into 5-μm sections perpendicular to the sagittal suture with a microtome (Leica). The TRAP solution was mixed with acetate buffer (pH 5.0), Fast Red Violet LB Salt (Sigma-Aldrich), naphthol AS-MX phosphate (Sigma-Aldrich), and 50 mM sodium tartrate to stain the paraffin sections. Hematoxylin was utilized to counterstain all sections. If a cell was TRAP-positive multinucleated cell, it was classified as an osteoclast. TRAP-positive multinucleated cells were counted as osteoclasts on the surface area of each section at the suture mesenchyme which lies on the sagittal suture of the mice as previously described (40).

### 2.4 μCT examination of area of bone destruction

After 5-day-subcutaneous supracalvarial injections, the mice were euthanized, and their calvariae were promptly fixed in 4% PBS-buffered formaldehyde. The calvariae were subsequently subjected to micro-computed tomography (μCT) (ScanXmate-E090; Comscan, Kanagawa, Japan) to produce reconstructed images using the TRI/3DBON64 software (RATOC System Engineering, Tokyo, Japan). The resorption area, distinguishable by the black appearance between the sutures, was identified against the white bone surface. To calculate the proportion of the bone destruction area to the entire area, a 7050-pixel rectangular region was drawn at the intersection of the sagittal and coronal sutures using ImageJ software (NIH, Bethesda, MD, USA) (40).

### 2.5 Preparation of osteoclast precursors for osteoclastogenesis

To obtain osteoclast precursors from mouse bone marrow cells, the lower limb long bones of male C57BL/6 sacrificed mice were excised. Bone marrow cells were extracted from femurs and tibiae using α-Minimum Essential Medium (α-MEM) from Sigma-Aldrich. The cell suspension was centrifuged twice at 4°C after being strained through a 40 μm nylon cell strainer (Falcon, NY, USA). The cells were then seeded in a 10 cm culture dish and cultured in 10 mL α-MEM supplemented with 10% fetal bovine serum (FBS), 100 IU/mL penicillin G (Meiji Seika, Tokyo, Japan), and 100 µg/mL streptomycin (Meiji Seika). Bone marrow cells were treated with 100 ng/mL M-CSF for 3 days to produce M-CSF-dependent macrophages. Washing with PBS removed non-adherent cells, and trypsin-EDTA solution was used to harvest adherent cells (Sigma-Aldrich). Adherent cells were used in this research as osteoclast precursors, as was previously described. Osteoclast precursors were plated separately in each well with the number of 4 × 10^4^ cells in a 96-well plate and cultivated in medium stimulated by M-CSF-only (100 ng/mL), M-CSF (100 ng/mL) and RANKL (100 ng/mL) or TNF-α (100 ng/mL) with different concentrations (0,0.01, 0.1, 1, or 10 μM) of azilsartan for 5 days. Discard the supernatant and the cells were then fixed in 10% formalin. After fixation, the cells were permeabilized for 30 min at ambient temperature with 0.2% Triton X-100 before incubation in a TRAP staining solution prepared as previously described. Under a light microscope, osteoclasts were identified as TRAP-positive multinucleated cells and counted.

### 2.6 Cell viability analysis for osteoclast precursors

In a 96-well plate, 1×10^4^ osteoclast precursors were seeded and M-CSF (100 ng/mL) and various azilsartan concentrations (0,0.01, 0.1, 1, or 10 μM) were added during the incubation process. After 5 d of incubation, the cells were cultured in each well with 200 μL of culture medium. Four replicates were analyzed for each sample. The plate was then incubated for 2 hours at 37°C with a 10 μL cell counting kit8 (Dojin, Kumamoto, Japan) solution in each well. Absorbance was measured at 450 nm using a microplate reader.

### 2.7 Peritoneal macrophages isolation

Macrophages were extracted from the peritoneal cavities of 8–10-week-old mice. Sterile ice-cold PBS (pH 7.4) 6mL injection was treated into the peritoneal cavity, and the fluid was aspirated to collect peritoneal cells and obtain resident macrophages under resting conditions. The cell suspension was centrifuged twice at 4°C after being strained through a 40 μm nylon cell strainer. Peritoneal cells were cultured in a 12-well plate for 2 h in the medium and washed twice to remove non-adherent cells. After a 24-hour incubation period, adherent cells were collected and used as macrophages (40).

### 2.8 Preparation of osteoblasts

The calvariae of five to six-day-old mice were dissected, and a collagenase solution (0.2% w/v) was prepared in an isolation buffer (3 mM K_2_HPO_4_, 10 mM NaHCO_3_, 60 mM sorbitol, 70 mM NaCl, 1 mM CaCl_2_, 0.5% [w/v] glucose, 0.1% [w/v] bovine serum albumin [BSA], and 25 mM 4-(2-hydroxyethyl)-1-piperazineethanesulfonic acid). A 0.2-μm filter was used to prepare EDTA at a concentration of 5 mM with 0.1% BSA in PBS. For 20 and 15 min, respectively, at 37°C on a shaker, the calvariae were exposed to digestion with collagenase and EDTA. Fractions 1 (collagenase), 2 (EDTA), 3 (collagenase), 4 (collagenase), and 5 (EDTA) and their digests were collected. The highest fractions for osteoblasts were regarded as fractions 3-5. The cells were cultured overnight in the medium. Trypsin-EDTA was used to harvest adherent cells, which were then cultivated for three days, and the medium was changed every two days. The adhered cells serve as osteoblasts (41).

### 2.9 RNA preparation and real-time RT-PCR analysis

To extract RNA from *in vivo* samples, liquid nitrogen was used to freeze the calvariae to extract RNA from the *in vivo* samples. Subsequently, they were pulverized using a Micro Smash MS-100R (Tomy Medico, Tokyo, Japan) in the presence of 800 μL of TRIzol reagent (Invitrogen, Carlsbad, CA). Total RNA was isolated using a RNeasy Mini Kit (Qiagen, Valencia, CA, USA). *In vitro* experiments involved the culture of osteoblasts or macrophages in a medium consisting of PBS, LPS alone (100 ng/mL), LPS (100 ng/mL) plus azilsartan (1 μM), or azilsartan (1 μM) alone. Total cellular RNA was extracted after 3 days of culture using the RNeasy Mini Kit. Reverse transcription of each RNA sample was performed using oligo(dT) primers (Invitrogen) and reverse transcriptase, and the resulting cDNA was quantified for Cathepsin K, TRAP, RANKL, and TNF-α mRNA transcripts using real-time RT-PCR with a Thermal Cycler Dice Real-Time System (Takara Bio, Shiga, Japan). The reaction mixture comprised 2 μL of cDNA template, 23 μL of SYBR^®^ Premix Ex Taq (Takara), and 50 pmol/μL of each primer in a total volume of 25 μL. The following primers were used in this study: 5′-GGTGGAGCCAAAAGGGTCA-3′ and 5′-GGGGGCTAAGCAGTTGGT-3′ for GAPDH; 5′-CTGTAGCCCACGTCGTAGC-3′ and 5′-TTGAGATCCATGCCGTTG-3′ for TNF-α; 5′-CCTGAGGCCCAGCCATTT-3′ and 5′-CTTGGCCCAGCCTCGAT-3′ for RANKL; 5′-GCAGAGGTGTGTACTATGA-3′ and 5′-GCAGGCGTTGTTCTTATT-3′ for cathepsin K; 5′-AACTTGCGACCATTGTTA-3′ and 5′-GGGGACCTTTCGTTGATGT-3′ for TRAP. Normalization of the expression levels of Cathepsin K, TRAP, RANKL and TNF-α mRNA transcripts was performed using the levels of GAPDH, which encodes for glyceraldehyde 3-phosphate dehydrogenase as a reference gene.

### 2.10 ELISA assay for TNF-α and RANKL

After 3-day culture of peritoneal macrophages and osteoblasts, cell supernatants were obtained respectively. TNF-α concentration was measured using Mouse TNF-alpha Sandwich ELISA Kit (KE10002, Proteintech, IL, USA) according to the manufacturer’s protocols. Added 100 μL of each standard and sample and incubate the plate for 2 hours at 37°C. Wash the wells four times with 1X Wash Buffer. After the last wash, added 100 μL of 1X Detection Antibody solution incubate for 1 hour at 37°C. The next step was adding 100 μL of 1X Streptavidin-HRP solution to each well. and incubate for 40 minutes at 37°C. For signal development, 100 μL of TMB substrate solution was added, protecting it from light for 20 minutes. Finally, adding 100 μL of Stop Solution to each well and read the results immediately at a wavelength of 450 nm using a microplate reader (Remote Sunrise, Tecan, Kawasaki, Japan). RANKL concentration was measured using RANKL (TNFSF11) Mouse ELISA kit (ab100749, Abcam, Cambridge, UK). Added standard or sample to each well and incubate 2.5 h at room temperature with gentle shaking. After the incubation period, discard the solution and wash the wells four times and added the prepared biotin antibody then incubate for 1 h. Next, the prepared HRP-Streptavidin solution was added, and it was let to sit at room temperature for 45 min. after that, proceed by adding 100 μL of TMB One-Step Substrate Reagent to each well and incubating for 0.5 h in the dark with gentle shaking. Then, use a microplate reader to measure the absorbance at 450 nm after adding 50 μL of Stop Solution.

### 2.11 Western blotting analysis

How azilsartan effects LPS-triggered phosphorylation of ERK, P38 and JNK mitogen-activated protein kinases (MAPKs) in macrophages was investigated. Macrophages were cultivated in a 6-well plate overnight in α-MEM supplemented with 10% FBS, 100 IU/ml penicillin G, 100 μg/ml streptomycin. Then, macrophages were cultured in α-MEM that was serum-free for starvation for 3 hours. The wells received additions of 100 ng/ml LPS only or LPS with azilsartan for durations of 0, 5, 15, and 30 minutes. LPS and azilsartan were absent from control wells (0 min). Insoluble material was removed by centrifugation after macrophages were lysed using radioimmunoprecipitation (RIPA) assay buffer (Millipore, MA, USA) containing 1% protease and phosphatase inhibitor (Thermo Fisher Scientific, IL, USA). In order to prepare SDS-PAGE gel electrophoresis, the protein was treated with β-Mercaptoethanol (BioRad, CA, USA) and Laemmli sample buffer (BioRad, CA, USA) 3:1 and denatured at 95°C for 5 min. Equal amounts of protein were loaded into gels 4–15% Mini-PROTEAN TGX Precast Gels (Bio-Rad, CA, USA) and transferred to a Trans-Blot Turbo Transfer System (Bio-Rad, CA, USA) and then incubated in Block-Ace (DS Pharma Biomedical, Osaka, Japan) 2 h at room temperature. Membranes were incubated with the following antibodies: Phospho-p38 MAPK (Thr180/Tyr182), p38 MAPK rabbit Ab, Phospho-p44/42 (ERK1/2) MAPK (Thr202/Tyr204), p44/42 (ERK1/2) MAPK rabbit Ab Phospho-SAPK/JNK (Thr183/Tyr185), SAP/JNK MAPK rabbit Ab (monoclonal rabbit IgG, 1:3000, Cell Signaling Technologies, MA, USA), anti-β-actin antibody (monoclonal mouse IgG, 1:5,000, Sigma-Aldrich, MO, USA) overnight at 4°C. The membranes were incubated with horseradish peroxidase-conjugated anti-rabbit antibody (Cell Signaling Technologies, MA, USA) at a dilution of 1:5,000 or anti-mouse antibody (GE Healthcare, IL, USA) at a dilution of 1:10,000 for 1 h at room temperature after being washed in tris-buffered saline with Triton X-100 (TBS-T). Bound antibodies were detected with SuperSignalWest Femto Maximum Sensitivity Substrate (Thermo Fisher Scientific, IL, USA) and a FUSION-FX6. EDGE Chemiluminescence Imaging System (Vilber Lourmat, Collégien, France).

### 2.12 Statistical analysis

Data are presented as mean values accompanied by their corresponding standard deviations. Statistical analysis was performed utilizing the Tukey–Kramer test and t-test to assess the significance of group differences. Statistical significance was set at P < 0.05 significance.

## 3 Results

### 3.1 Azilsartan inhibited LPS-triggered osteoclastogenesis *in vivo*

**Figure 1:**
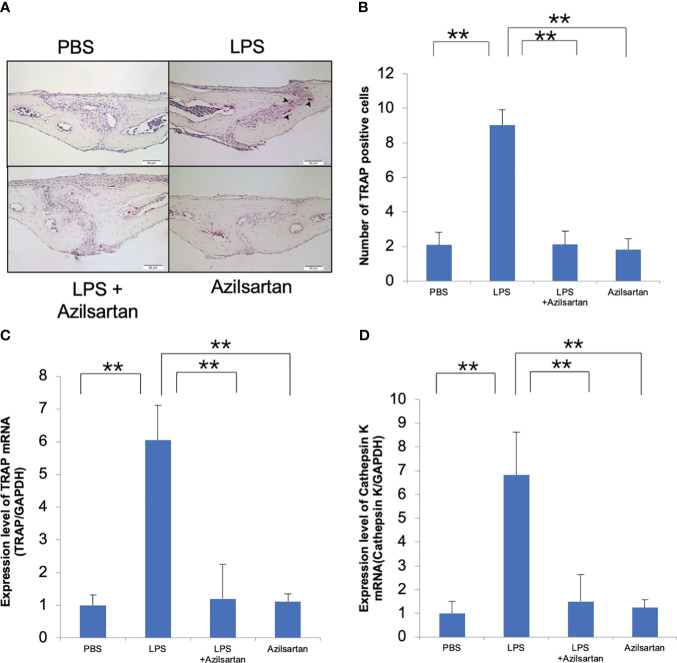
Azilsartan restrained LPS-triggered osteoclastogenesis *in vivo.* **(A)** TRAP-stained histological sections of mice calvariae, which were utilized to distinguish osteoclasts, were obtained after a 5-day consecutive subdermal injection with PBS, LPS (100 µg/day) in the presence or absence of azilsartan (100 µg/day), and azilsartan (100 µg/day) alone. Hematoxylin was utilized to counterstain all sections. **(B)** The number of TRAP-positive multinucleated cells in the sagittal suture mesenchyme of the calvaria was determined. Scale bar = 50 μm. **(C)** The mRNA levels of TRAP transcripts in mice calvariae were quantified. **(D)** The mRNA levels of Cathepsin K in mice calvariae were quantified. The Tukey–Kramer test was used to assess the significance of group differences. Values are reported as means ± SD (n =4/group, ∗∗p <0.01).

The effect of azilsartan on LPS-triggered osteoclast formation in mouse calvaria was investigated. To achieve this, LPS was administered to mice either alone or in combination with azilsartan, and the effects were observed in histological sections. After 5 consecutive days of LPS administration, numerous large TRAP-positive multinucleated cells were observed inside the suture mesenchyme. Compared with PBS and azilsartan administration groups, the number of osteoclasts was more in the LPS group. However, the results indicated that the average number of osteoclasts was considerably lower in the group that received both LPS and azilsartan than that in the group that received LPS alone (**Figures 1A, B**). In addition, the LPS group displayed higher TRAP and cathepsin K mRNA levels than PBS and azilsartan groups. Mice administered LPS with azilsartan showed considerably lower levels of TRAP and cathepsin K mRNA than mice administered LPS alone (**Figures 1C, D**).

### 3.2 Azilsartan inhibited LPS-triggered bone resorption *in vivo*

**Figure 2:**
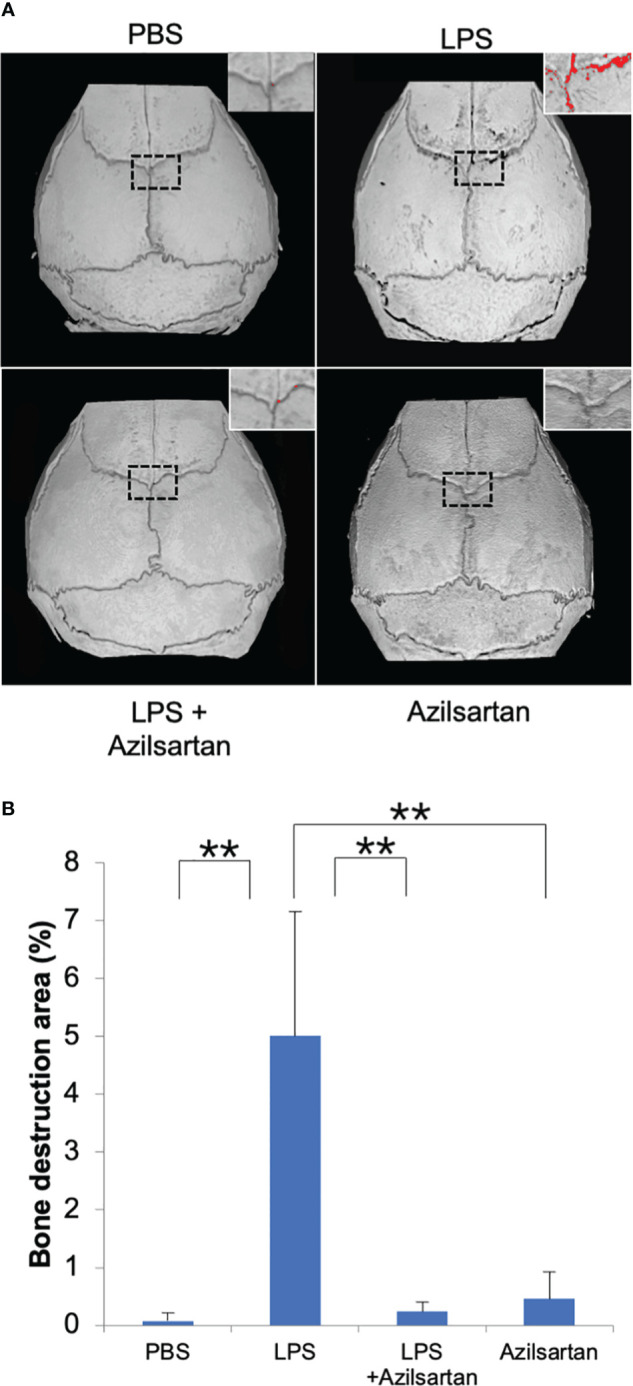
Azilsartan restrained LPS-triggered bone resorption *in vivo*. **(A)** μCT reconstruction of calvariae. The mice received subdermal injections of the calvariae with PBS, LPS (100 µg/day) in the presence or absence of azilsartan (100 µg/day), and azilsartan alone (100 µg/day) for 5 consecutive days, and calvariae were excised on the sixth day and promptly fixed in 4% PBS-buffered formaldehyde. Then mice calvariae were scanned by micro-computed tomography to produce reconstructed images. The red dots indicate areas of bone resorption. **(B)** The proportion of bone resorption area to the entire bone area. Tukey–Kramer test is utilized to assess the significance of group differences. Values are reported means ± SD (n =4/group; ∗∗p <0.01).

Following μCT scanning, the proportion of bone destruction area to the entire calvarial area was analyzed in calvaria. Results indicated that the LPS group exhibited a significantly greater bone resorption area than the PBS and azilsartan groups. Co-administration of LPS and azilsartan led to a dramatic decrease in the bone resorption area compared to that in the LPS group (**Figures 2A, B**).

### 3.3 Azilsartan had inhibitory impact on expression of oteoclast-related cytokines (RANKL and TNF-α) *in vivo*

**Figure 3:**
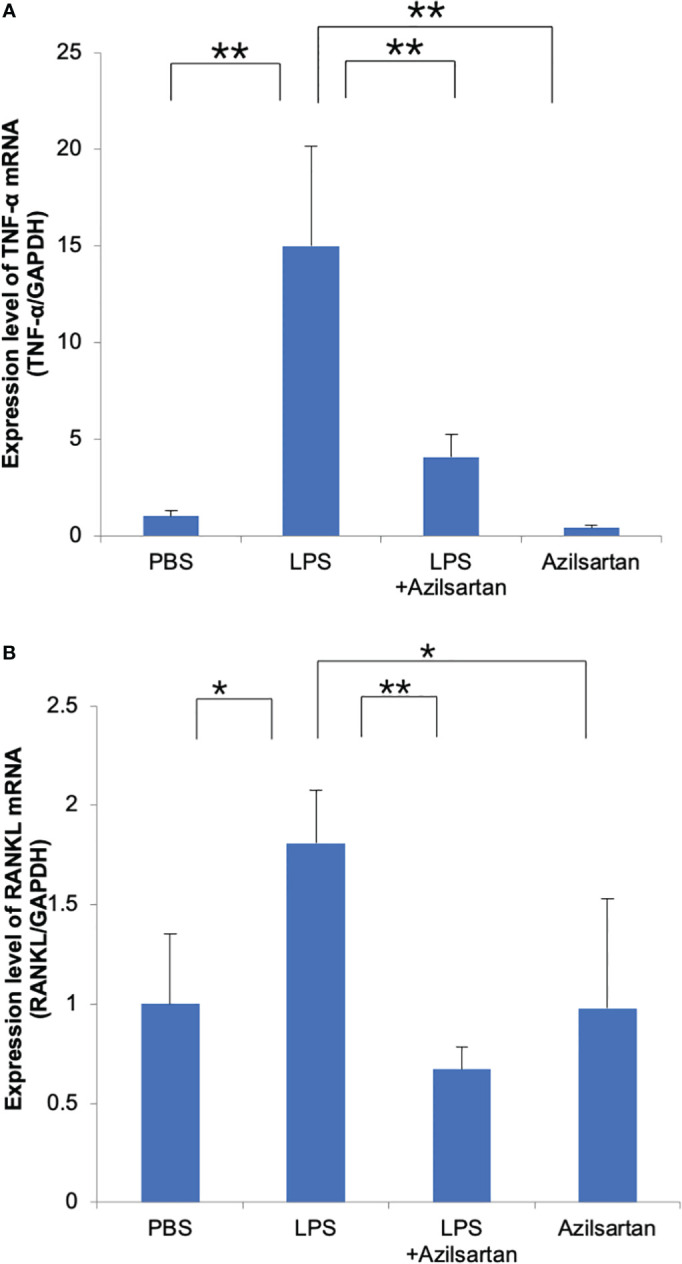
Azilsartan restrained LPS-triggered expression of TNF-α and RANKL *in vivo*. Total RNA was extracted from mice calvariae after 5 days of subcutaneous injections with PBS, LPS (100 μg/day) with or without azilsartan (100 μg/day), and azilsartan alone (100 μg/day). **(A)** TNF-α mRNA levels in mice calvariae were assessed. **(B)** RANKL mRNA levels in mice calvariae were assessed. Tukey–Kramer test is utilized to assess the significance of group differences. Values are reported as means ± SD. (n =4/group; ∗p <0.05, ∗∗p <0.01).

The mRNA expression levels of osteoclast-related cytokines (TNF-α and RANKL) in mouse calvarial bone chips were assessed. Compared with the PBS-and azilsartan-treated groups, the LPS-administered group displayed increased TNF-α and RANKL mRNA levels. In contrast to the LPS-only group, the azilsartan and LPS co-administered group had lower levels of TNF-α and RANKL mRNA expression (**Figures 3A, B**).

### 3.4 Azilsartan had no impact on cell viability of osteoclast precursors, RANKL or TNF-α−triggered osteoclast formation

**Figure 4:**
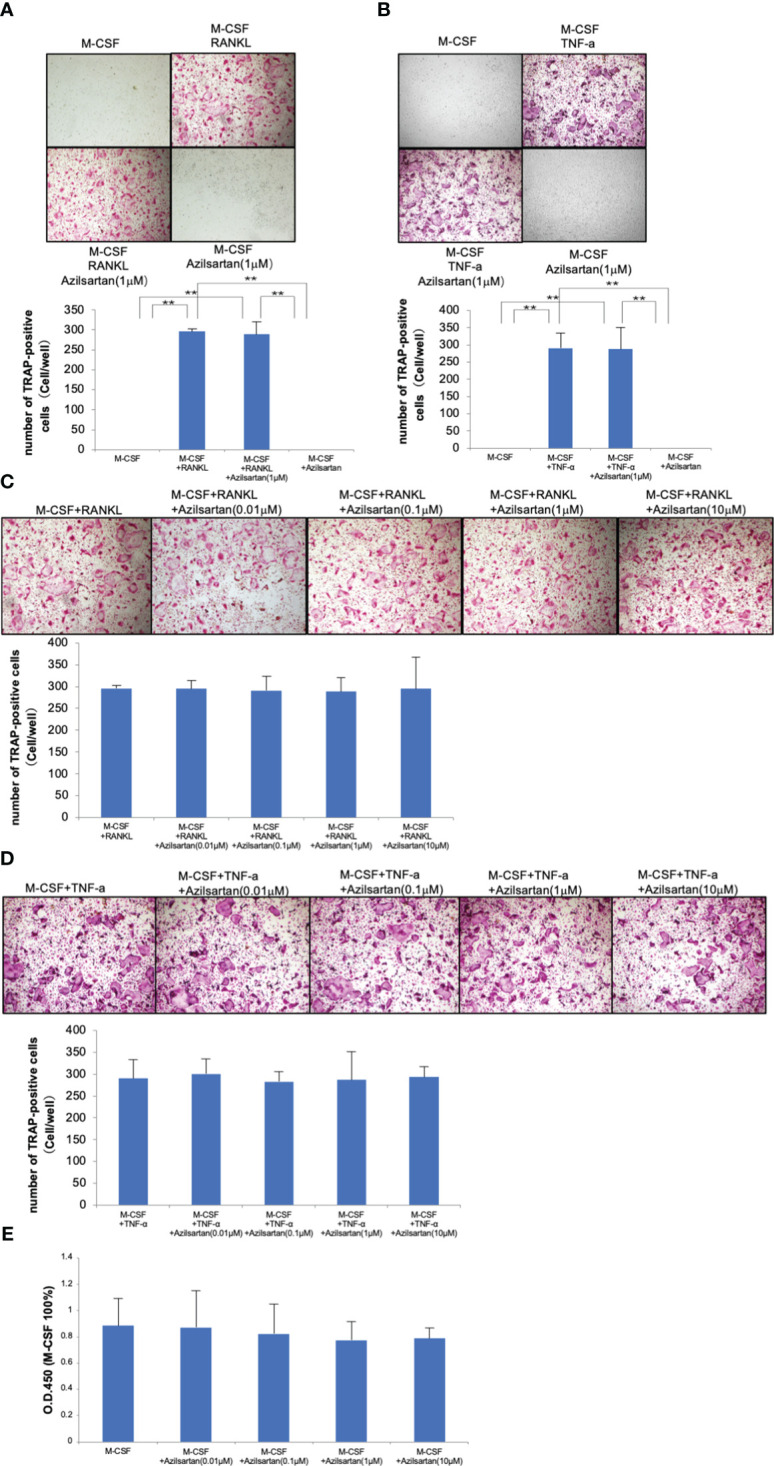
Azilsartan did not impact on RANKL-triggered osteoclast generation, TNF-α-triggered osteoclast generation, and osteoclast precursor cell viability *in vitro.* Mouse bone marrow cells were extracted from femurs and tibiae using Bone marrow cells were treated with 100 ng/mL M-CSF for 3 days to produce M-CSF-dependent macrophages. Adherent cells were used in this research as osteoclast precursors. **(A)** Micrographs and quantity of TRAP-positive multinucleated cells. Osteoclast precursor cells were cultivated with M-CSF, M-CSF adding RANKL with or without the presence of azilsartan and azilsartan alone, followed by TRAP staining. **(B)** Micrographs and quantity of TRAP-positive multinucleated cells. Osteoclast precursor cells were cultured with M-CSF, M-CSF adding TNF-α with or without the presence of azilsartan and azilsartan alone, followed by TRAP staining. **(C)** Micrographs and quantity of TRAP-positive multinucleated cells. Osteoclast precursor cells were cultivated with M-CSF adding RANKL with various concentrations of azilsartan (0, 0.01, 0.1, 1, or 10 μM), followed by TRAP staining. **(D)** Micrographs and quantity of TRAP-positive multinucleated cells. Osteoclast precursors were cultured with M-CSF adding RANKL with various concentrations of azilsartan (0, 0.01, 0.1, 1, or 10 μM), followed by TRAP staining. **(E)** Cell viability of osteoclast precursors treated with M-CSF alone and M-CSF with various concentrations of azilsartan (0, 0.01, 0.1, 1, or 10 μM) for 5 days. CCK-8 assay was utilized to evaluate cell viability. Data are presented as percentage activity relative to the activity in the cultivation with M-CSF alone. Tukey–Kramer test is utilized to assess the significance of group differences. Values are reported as means ± SD (n =4/group; ∗∗p <0.01).

The influence of various azilsartan concentrations (0, 0.01, 0.1, 1, and 10 μM) on RANKL-triggered osteoclastogenesis, TNF-α-induced osteoclastogenesis, and osteoclast precursor cell viability were examined to investigate the direct effects of azilsartan on osteoclast precursor cells. Many TRAP-positive cells were seen in osteoclast precursor cells cultured with M-CSF, RANKL or TNF-α, as well as in osteoclast precursor cells cultured with M-CSF, RANKL, or TNF-α in the presence of azilsartan (**Figures 4A–D**). In addition, after five consecutive days of culture, there was no obvious change in cell viability among the various azilsartan concentrations (0, 0.01, 0.1, 1, and 10 μM) (**Figure 4E**).

### 3.5 Azilsartan suppressed LPS-triggered TNF-α expression in macrophages and had no effect on LPS-triggered RANKL expression in osteoblasts

**Figure 5:**
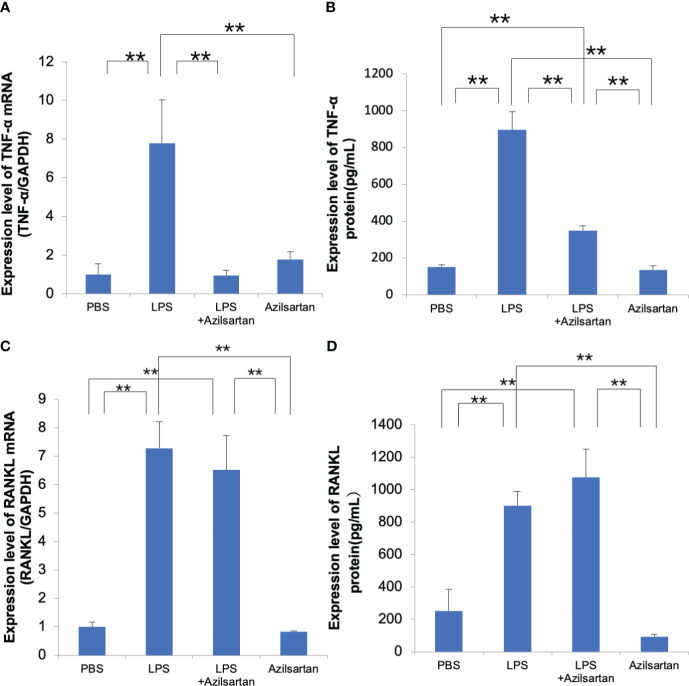
Azilsartan restrained LPS-triggered expression of TNF-α in macrophages and had no effect on LPS-triggered RANKL expression in osteoblasts **(A)** Cells were extracted from the peritoneal cavities of 8–10-week-old mice and adherent cells were used as macrophages. TNF-α mRNA levels in macrophages were assessed. Total RNA was extracted from macrophages cultivated with PBS, LPS in the presence or absence of azilsartan, and azilsartan only. **(B)** TNF-α protein levels in macrophages were assessed. Protein was extracted from macrophages cultivated with PBS, LPS in the presence or absence of azilsartan, and azilsartan only. **(C)** The calvariae of five to six-day-old mice were dissected. Fractions 1 (collagenase), 2 (EDTA), 3 (collagenase), 4 (collagenase), and 5 (EDTA) and their digests were collected. The highest fractions for osteoblasts were regarded as fractions 3-5. The adhered cells serve as osteoblasts. RANKL mRNA expression levels in osteoblasts were evaluated. Total RNA was isolated from osteoblasts cultivated with PBS, LPS in the presence or absence of azilsartan, and azilsartan only. **(D)** RANKL protein expression levels in osteoblasts were evaluated. Protein was isolated from osteoblasts cultivated with PBS, LPS in the presence or absence of azilsartan, and azilsartan only. Tukey–Kramer test is utilized to assess the significance of group differences. Values are reported as means ± SD (n =4/group; ∗∗p <0.01).

Real-time RT-PCR was applied to assess the levels of TNF-α mRNA expression in Macrophages. Compared with the PBS and azilsartan groups, the LPS group displayed increased TNF-α mRNA levels. Compared to the LPS-only group, the LPS with azilsartan group showed lower levels of TNF-α mRNA expression (**Figure 5A**). We further detected the TNF-α protein expression. As expected, LPS upregulated TNF-α protein expression but LPS with azilsartan group showed lower levels of TNF-α protein expression (**Figure 5B**). Furthermore, RANKL mRNA levels in osteoblasts were analyzed. Compared with the PBS and azilsartan group, the LPS group displayed increased RANKL mRNA levels. However, both LPS- and azilsartan-treated osteoblasts showed RANKL mRNA expression levels comparable to those in osteoblasts treated with LPS alone (**Figure 5C**) We also determined the RANKL protein expression. As expected, the protein expression of RANKL trend was consistent with the mRNA expression level (**Figure 5D**).

### 3.6 Azilsartan suppressed LPS-triggered MAPKs signaling pathway in macrophages

**Figure 6:**
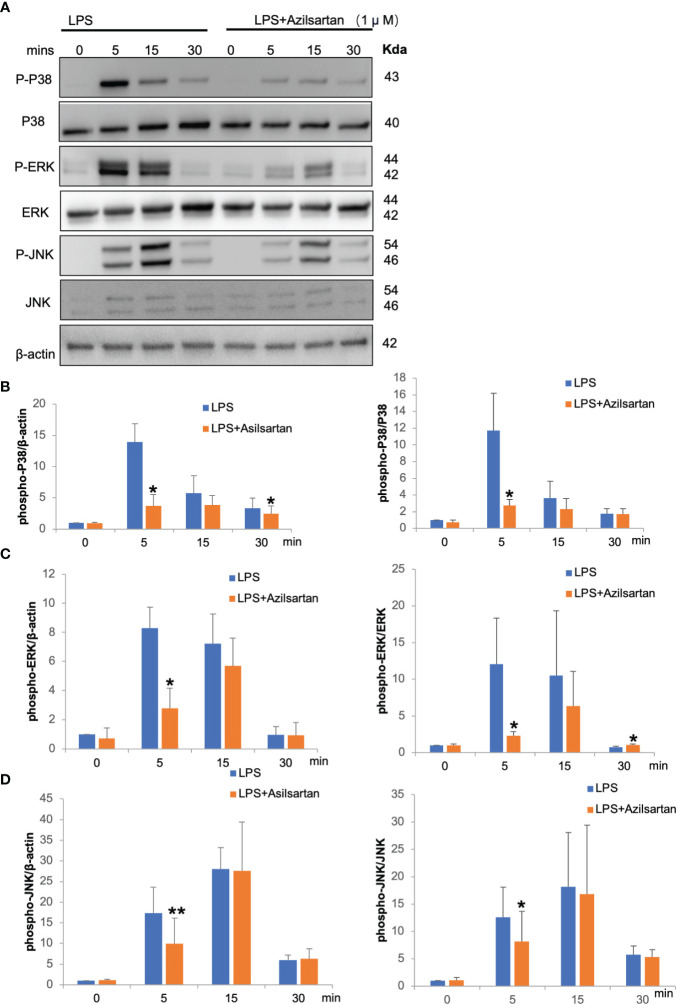
Azilsartan inhibited LPS-triggered activation of MAPK pathways *in vitro*. **(A)** Cells were extracted from the peritoneal cavities of 8–10-week-old mice and adherent cells were used as macrophages which were starved for 3 (h) Then, LPS with or without azilsartan was added for specific periods (0, 5, 15 and 30 min). Proteins were collected and the P38/ERK/JNK, phospho-P38/ERK/JNK and β-actin were detected by Western Blotting. **(B)** Analysis of the phospho-P38 band intensity in relation to β-actin and P38 quantitatively. **(C)** Analysis of the phospho-ERK band intensity in relation to β-actin and ERK quantitatively. **(D)** Analysis of the phospho-JNK band intensity in relation to β-actin and JNK quantitatively. T-test is utilized to assess the significance of group differences. Values are reported as means ± SD. (n =3/group; *p < 0.05, **p < 0.01).

LPS and LPS with azilsartan were added to cultures of macrophages at the indicated time points (0, 5, 15 and 30 min), while 0 indicates that there was no LPS or azilsartan. LPS temporarily increased the phospho-ERK/P38/JNK in relation to the corresponding total protein and β-actin, peaking at 5 or 15 minutes (**Figure 6A**). And azilsartan suppressed LPS-triggered phosphorylation of all three kinases after just 5 min of incubation (**Figures 6B–D**).

### 3.7 Azilsartan had no impact on TNF-α−triggered osteoclastogenesis *in vivo*

**Figure 7:**
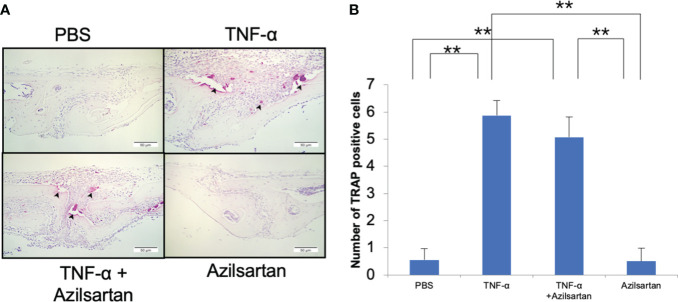
Azilsartan had no impact on TNF-α-triggered osteoclastogenesis *in vivo.* **(A)** TRAP-stained histological sections of mice calvariae, which were utilized to distinguish osteoclasts, were obtained after a 5-day consecutive subdermal injection with PBS, TNF-α (3 µg/day) in the presence or absence of azilsartan (100 µg/day), and azilsartan (100 µg/day) alone. Hematoxylin was utilized to counterstain all sections. **(B)** The number of TRAP-positive multinucleated cells in the sagittal suture mesenchyme of the calvaria was determined. Scale bar = 50 μm. The Tukey–Kramer test was used to assess the significance of group differences. Values are reported as means ± SD (n =4/group, ∗∗p <0.01).

The effect of azilsartan on TNF-α-triggered osteoclast formation in mouse calvaria was investigated. To achieve this, TNF-α was administered to mice either alone or in combination with azilsartan, and the effects were observed in histological sections. After 5 consecutive days of TNF-α administration, numerous large TRAP-positive multinucleated cells were observed inside the suture mesenchyme. Compared with PBS and azilsartan administration groups, the number of osteoclasts was more in the TNF-α group. And there is no difference between TNF-α group and TNF-α with azilsartan group (**Figures 7A, B**).

## 4 Discussion

Our goal was to determine the potential of azilsartan, an ARB, to regulate LPS-triggered osteoclastogenesis and bone resorption *in vivo*. Our results revealed that treatment with azilsartan effectively inhibited LPS-triggered osteoclastogenesis and bone resorption while inhibiting RANKL and TNF-α expression *in vivo*. However, azilsartan did not directly inhibit RANKL-triggered osteoclastogenesis, TNF-α-triggered osteoclastogenesis, or osteoclast precursor cell viability *in vitro*. Furthermore, azilsartan did not significantly suppress LPS-triggered RANKL expression in osteoblasts *in vitro*. However, azilsartan effectively inhibited the LPS-triggered TNF-α expression in macrophages *in vitro*.

As lifestyles continue to shift towards more sedentary and unhealthy patterns, the prevalence of hypertension is increasing. A growing number of people consume diets high in sodium and saturated fats while engaging in little physical activity. These lifestyle factors, combined with genetic predispositions and other medical conditions, contribute to the development of hypertension. As the world’s population continues to age and grow, the burden of hypertension is expected to increase, placing significant strain on healthcare systems and economies (1–3). Recent studies have demonstrated a connection between hypertension and a higher risk of osteoporosis and bone fractures, which may be mediated by the effects of the RAS on bone metabolism. Research has linked bone remodeling in osteoporosis to the renin-angiotensin system (39). The classical RAS axis involves ACE/ANG II/AT1R, which accounts for vasoconstriction and proinflammatory effects. Specifically, ANG II, a key component of RAS, stimulates osteoclastogenesis and bone resorption through the activation of AT1R (42–45). AT1R has been identified in osteoclast precursors and mature osteoclasts, indicating that the RAS may directly impact bone metabolism (46). In addition, several studies have shown that blocking AT1R with drugs can have beneficial effects on bone metabolism, potentially by reducing osteoclastogenesis and bone resorption (47–49).

Our study investigated the effects of azilsartan on LPS-triggered osteoclast formation in mice calvariae. After 5 days of subcutaneous injection of 100 μg LPS with or without azilsartan into mice calvariae, we analyzed the effects by histological sections. The results showed that azilsartan significantly inhibited osteoclast formation triggered by LPS, as evidenced by the lower mean number of osteoclasts. Real-time RT-PCR was utilized to assess the levels of TRAP and cathepsin K mRNA in mice and the group administered both LPS and azilsartan had lower levels of TRAP and cathepsin K mRNA than those administered LPS alone. Our study used μCT scanning to analyze the proportion of the bone resorption area to the entire calvarial area in mice. The results showed that mice treated with LPS and azilsartan had significantly smaller bone resorption areas than mice treated with LPS alone. This finding is in line with previous studies that showed the potential of azilsartan to restrain osteoclastogenesis and bone resorption. For instance, Pan et al. showed that azilsartan administration prevented OVX-Induced Bone Loss in mice (36). Similarly, another study by Araújo et al. found that azilsartan successfully reduced bone loss in rats with ligature-induced periodontitis (37).

We tried to investigate the role of AT1R in the mechanism underlying the effects of azilsartan on LPS-triggered osteoclastogenesis. To assess the involvement of AT1R, after 5 days of subcutaneous injection of 100 μg LPS with or without azilsartan into mice calvariae, we evaluated the expression of AT1R mRNA levels *in vivo*. Our results demonstrated there was no difference between PBS, LPS, LPS with azilsartan and azilsartan-only group (**Supplementary Figure 1A**). Earlier research showed administration of LPS to hypertensive mice cannot increase AT1R expression in cortex region and increased in hippocampus region (50). Previous studies reported that expression of AT1R in LPS-untreated and treated THP-1 macrophages at the protein level have no difference (51). Another study demonstrated that LPS may inhibit the expression of AT1R mRNA level in TI cells (52). Also, Ye et al. showed that LPS increased the expression of AT1R mRNA level in BEAS-2B cells (53). Therefore, to further validate our findings, we assessed the expression of AT1R mRNA level in macrophages, which yielded consistent findings (**Supplementary Figure 1B**). These observations highlight the complexity and context-dependent nature of AT1R regulation and its response to various stimuli. And azilsartan may suppress LPS-triggered osteoclastogenesis by other mechanisms rather than decreasing AT1R.

In previous reports, LPS induce phosphorylation of p38, ERK and JNK in macrophages. When MAPKs were inhibited by several inhibitors, expression of TNF-α was inhibited (54–56). In the present study, LPS also induced phosphorylation of p38, ERK and JNK in macrophages. The obtained results from the western blotting analysis indicate that azilsartan treatment significantly inhibits MAPKs signaling, including the phosphorylation of P38, ERK, and JNK in macrophages. We showed that azilsartan inhibited LPS-induced TNF-α expression in macrophages. These results suggested that azilsartan may inhibit TNF-α expression via suppression of LPS-induced phosphorylation of p38, ERK and JNK in macrophages.

**Figure 8:**
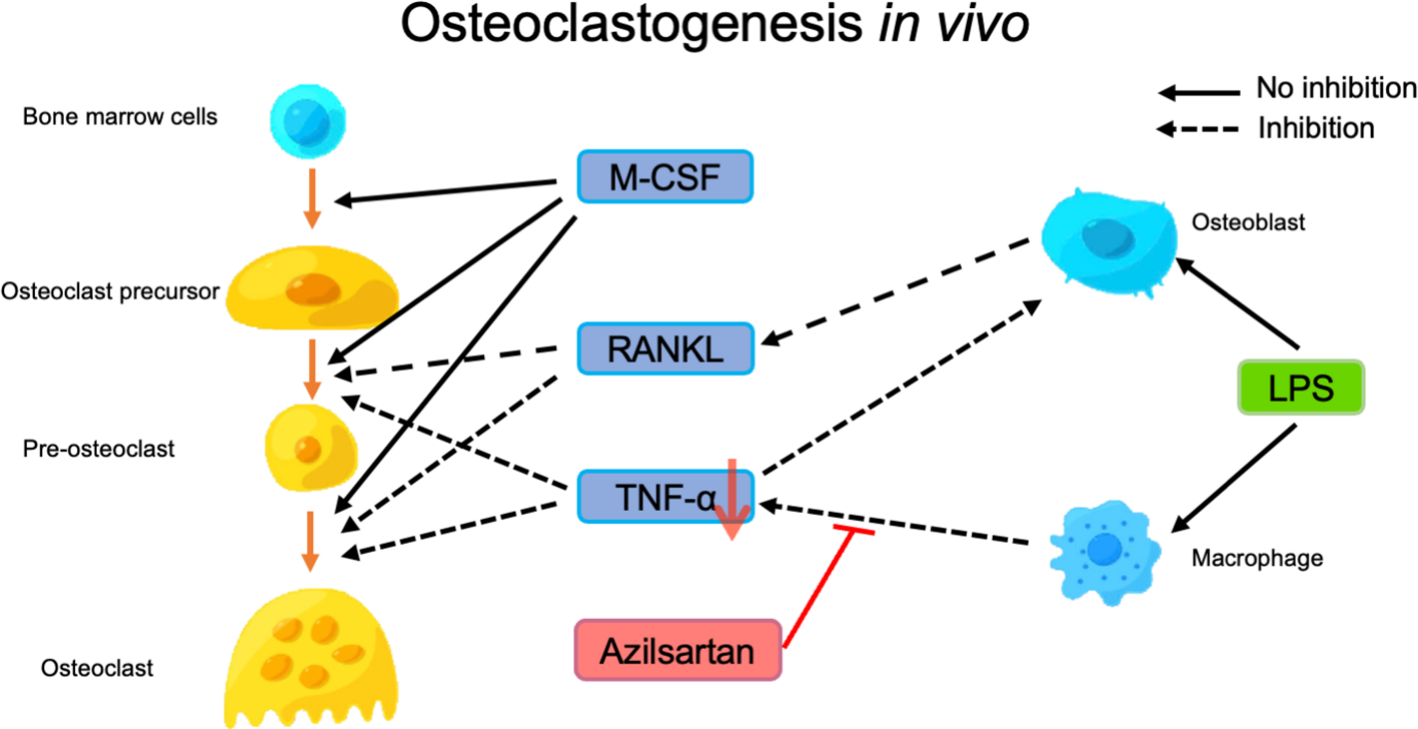
Putative model of how azilsartan may play a role in preventing LPS-triggered osteoclastogenesis *in vivo*. Osteoclast differentiation is triggered by RANKL and TNF-α, with M-CSF promoting the differentiation of osteoclast precursors into pre-osteoclasts, which subsequently fuse to form osteoclasts and induce bone resorption. LPS administration induces the expression of RANKL in osteoblasts and TNF-α in macrophages, which contributes to osteoclast formation and bone resorption. Azilsartan appears to inhibit LPS-triggered osteoclastogenesis by reducing the production of TNF-α by macrophages, even though it does not significantly inhibit osteoclast precursors or RANKL expression in osteoblasts.

This study aimed to investigate the mechanisms underlying the suppression of LPS-triggered osteoclastogenesis and bone resorption by azilsartan. We evaluated two potential mechanisms and conducted in-depth experiments to explore them further. We examined whether azilsartan inhibits the expression of the inflammatory cytokines TNF-α and RANKL, which are known to promote osteoclast formation (9, 57). Our findings revealed that azilsartan significantly suppressed LPS-triggered increases in TNF-α and RANKL mRNA levels caused by LPS *in vivo*. This indicated that azilsartan effectively inhibited osteoclast formation by targeting these crucial cytokines. We investigated whether azilsartan directly inhibited osteoclast formation. However, despite its inhibitory effect on LPS-triggered osteoclastogenesis and bone resorption *in vivo*, azilsartan was found to have no significant inhibition on the differentiation of osteoclast precursor cells into mature osteoclasts triggered by RANKL or TNF-α. These results suggest that azilsartan may act through a different pathway or target a different aspect of the osteoclast differentiation process than RANKL or TNF-α signaling. After 5 days of culture, we found no difference in cell viability between the different concentrations of azilsartan. Therefore, our experiments showed that azilsartan did not directly affect the osteoclast precursors. After evaluating the effect of azilsartan on LPS-triggered RANKL expression in osteoblasts, we got the result that azilsartan did not affect LPS-triggered RANKL expression in osteoblasts. Next, we assessed whether azilsartan restrains LPS-triggered TNF-α mRNA expression in macrophages. Instead, we found that azilsartan restrained LPS-triggered TNF-α expression in macrophages, which plays a vital role in promoting RANKL expression and osteoclast formation. To provide a critical test to ascertain whether the suppression of bone loss by Azilsartan is primarily mediated through TNF-α inhibition, 3 μg TNF-α with or without azilsartan were injected into mice calvariae for 5 days, the result showed that azilsartan had no impact on TNF-α-triggered osteoclastogenesis *in vivo.* Therefore, our study suggests that the *in vivo* suppression of LPS-triggered osteoclastogenesis by azilsartan is possibly due to the suppression of TNF-α expression in macrophages, leading to the restraining of RANKL expression in stromal cells (**Figure 8**).

## 5 Conclusion

Overall, our study provides valuable insights into the mechanisms underlying the suppressive effects of azilsartan on osteoclast generation and bone resorption. This finding is important for individuals with hypertension, who are at an increased risk of osteoporosis and fractures.

## Data availability statement

The raw data supporting the conclusions of this article will be made available by the authors, without undue reservation.

## Ethics statement

The animal study was approved by The Science Animal Care and Use Committee of Tohoku University. The study was conducted in accordance with the local legislation and institutional requirements.

## Author contributions

Conceptualization: ZF and HK. Methodology: HK. Validation: ZF and HK. Formal analysis: ZF, FO, and TN. Investigation: ZF, HK, AM, FO, JM, KK, MM, JR, KN, and AL. Resources: HK. Data curation: ZF. Writing (original draft preparation): ZF. Writing (review and editing): HK. Supervision: IM. Project administration: HK. Funding acquisition: HK. All authors contributed to the article and approved the submitted version.
