# Supplementary material for: Azilsartan inhibits inflammation-triggered bone resorption and osteoclastogenesis in vivo via suppression of TNF-α expression in macrophages
Source: Front Endocrinol (Lausanne). 2023 Sep 15;14:1207502. doi: 10.3389/fendo.2023.1207502 (PMC10545845; doi:10.3389/fendo.2023.1207502)

**Supplement Figure 1.** Azilsartan had no impact on LPS-triggered AT1R expression *in* *vivo* and *in vitro*. (A) AT1R mRNA levels were assessed *in vivo*. AT1R mRNA levels in mouse calvariae were assessed. Total RNA was extracted from mice calvariae after 5 days of subcutaneous injections with PBS, LPS (100 μg/day) with or without azilsartan (100 μg/day), and azilsartan alone (100 μg/day). (B)AT1R mRNA levels in macrophages was assessed. Total RNA was extracted from macrophages cultivated with PBS, LPS in the presence or absence of azilsartan, and azilsartan only. Tukey–Kramer test is utilized to assess the significance of group differences. Values are reported as means ± SD. (n =4/group)
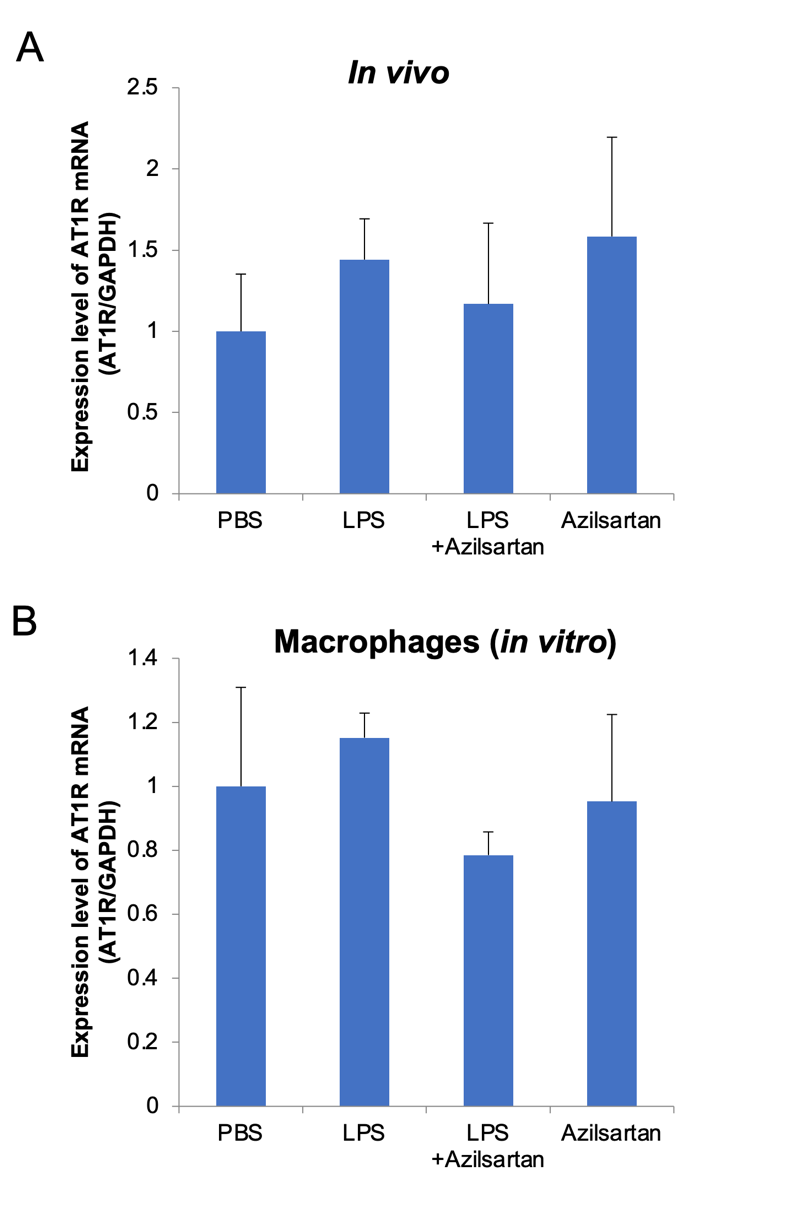

Supplement: Supplementary file 1 [file DataSheet_1.docx]
